# Language-Concordant Primary Care Physicians for a Diverse Population: The View from California

**DOI:** 10.1089/heq.2019.0035

**Published:** 2019-07-01

**Authors:** Maria E. Garcia, Andrew B. Bindman, Janet Coffman

**Affiliations:** ^1^ Division of General Internal Medicine, Department of Medicine, University of California at San Francisco (UCSF), San Francisco, California.; ^2^ Philip R. Lee Institute for Health Policy Studies, University of California at San Francisco (UCSF), San Francisco, California.; ^3^ Department of Family and Community Medicine, University of California, San Francisco (UCSF), San Francisco, California.

**Keywords:** immigrant health, primary care, limited English proficiency, bilingual physicians

## Abstract

**Purpose:** The population with limited English proficiency (LEP) in California is growing. We sought to determine whether enough primary care physicians (PCPs) have the language skills to meet patient needs.

**Methods:** The authors determined the number of PCPs who self-report proficiency in the five most common non-English languages spoken in California (Spanish, Cantonese, Mandarin, Tagalog, and Vietnamese) using Medical Board of California data from 2013 to 2015. The authors estimated LEP populations during 2011–2015 using Census data. They calculated PCP supply (the ratio of PCPs/100,000 LEP individuals) compared to a federal standard to judge adequacy. They performed a sensitivity analysis adjusting the percentage of LEP patients in a bilingual physicians' practice from 100% to the percentage of LEP individuals in California who spoke that language.

**Results:** Of 19,310 PCPs in California, 15,933 (83%) provided information about languages they speak. There were 5,203 (33%) Spanish-, 486 (3%) Cantonese-, 986 (6%) Mandarin-, 956 (6%) Tagalog-, and 671 (4%) Vietnamese-speaking PCPs. PCP supply, compared to a federal standard, was adequate if we assumed that bilingual PCPs only care for LEP patients. However, if one assumes the number of LEP patients in a PCP's practice reflects the percentage in the general population, there is a large PCP undersupply for all languages.

**Conclusion:** Estimates of access to language-concordant PCPs for LEP individuals are sensitive to assumptions about the percentage of LEP patients in a PCP's panel. Ensuring language-concordant access will require deliberate effort to match LEP patients with bilingual PCPs.

## Introduction

Sixty-four million Americans speak a language other than English at home and nearly 26 million of those report limited English proficiency (LEP)—defined as speaking English less than “very well.”^1^ These numbers are expected to grow over the next few decades.^2^ Language barriers between patients and their providers have been linked to a number of disparities, including less patient-centered care, decreased receipt of recommended preventive health services, diminished joint decision-making, poor patient-physician communication, and difficulties developing trust.^3–8^ Furthermore, language discordance can present additional barriers to health systems, as it is associated with increased interpreter costs, lower provider and patient satisfaction, and increased opportunities for medical errors.^5,7,9^ In contrast, language concordance (i.e., when patients and physicians speak the same language) is associated with improved quality of care, including improved control of chronic medical conditions, greater health education received, and improved adherence to medications, compared to reliance on professional interpreters.^4,10–12^

While the number of individuals with language barriers in the United States is growing, it is not known whether there are enough primary care physicians (PCPs) with proficiency in non-English languages to meet patient needs. Prior work in California found an inadequate number of Spanish-speaking PCPs when accounting for insurance status; few or no studies have focused on the other four most commonly spoken languages in the state (Cantonese, Mandarin, Tagalog, and Vietnamese) independently.^13–16^ A physician's race/ethnicity and whether they work in areas with high concentrations of individuals with LEP have been associated with non-English language proficiency.^14,15^ International medical graduates (IMGs) were also more likely to report fluency in a language other than English.^15^

California, with its large population, long history of immigration, and racial and linguistic diversity, is a good place to assess whether PCP language capacity matches population needs. As the population with LEP grows nationally, other states may learn from the California experience.

In this study, we applied several different assumptions regarding the percentage of language-concordant patients with LEP in self-reported bilingual PCPs' practices to determine the adequacy of PCP supply for patients with LEP compared to a federal standard. We further evaluated those factors (race/ethnicity and graduation from an international versus a U.S. medical school) that have been associated with self-reported proficiency in non-English languages to assess whether findings from our study are consistent with prior studies.

## Methods

We examined Medical Board of California data to determine the numbers of PCPs who self-report proficiency in Spanish, Cantonese, Mandarin, Tagalog, or Vietnamese. The Medical Board of California surveys all physicians every 2 years in conjunction with required renewal of their licenses to remain active in practice. The survey methods have been described previously in detail.^14–18^ In brief, all physicians complete a mandatory questionnaire on basic demographics, non-English language proficiency, weekly hours dedicated to direct patient care, research, teaching and administration, current training status, location of medical school, practice location (zip code of the primary practice location), and specialty. For this study, we restricted our analysis to PCPs (Family Medicine, Internal Medicine, General Practice, Geriatric Medicine, and General Pediatrics physicians) with a practice address in California, who renewed their licenses between September 2013 and August 2015, who were involved in direct patient care for 20 hours or more per week (per the American Medical Association's definition of active patient care physicians), who were no longer in training, and who responded to the question on non-English language proficiency.^18^ Given the license renewal period of every 2 years, the survey responses capture the vast majority of active PCPs practicing in California during this time period, with the exception that some may have died, relocated, or retired during the study period.

We included physicians who completed two voluntary items on race/ethnicity and language: (1) self-identified race/ethnicity from a detailed list of 37 race and ethnicities and (2) self-reported proficiency in any of 53 languages listed on the survey. The question on self-reported language proficiency is phrased as follows: “In addition to English, indicate additional languages in which you are proficient.” Physicians can skip questions or check more than one response to the race/ethnicity and language items. We limited our analysis to Spanish, Cantonese, Mandarin, Tagalog, and Vietnamese, the five most commonly spoken languages in California other than English.

We used estimates of the number of Californians with LEP from the 2011 to 2015 American Community Survey (ACS) Public Use Microdata Sample (PUMS) 5-year estimates.^19^ The ACS asks individuals who speak a non-English language to rate their English fluency with the following question, “How well do you speak English?” Response options include “very well,” “well,” “not well,” and “not at all.” Patients who spoke English less than “very well” are classified as LEP. We focused our analysis on individuals with LEP, as opposed to the whole population who report speaking a given non-English language, because we felt those individuals would most benefit from having a physician who spoke their non-English language. Even if English is not their preferred language, individuals fluent in English may not experience the same challenges as individuals who do not speak English very well.

We further categorized physicians as active in one of 10 regions in California based on zip code of practice location, as has been described in previous reports using these data.^20^ Federal standards established by the Council on Graduate Medical Education suggest the need for 60–80 PCPs per 100,000 individuals.^21^ We used this benchmark to evaluate the adequacy of PCP availability for Californians with LEP.

### Statistical analysis

We used the Medical Board of California data to estimate the number and percentage of PCPs who report proficiency in Spanish, Cantonese, Mandarin, Tagalog, or Vietnamese, according to physician demographics. We estimated total California population, Spanish speakers, Tagalog speakers, and Vietnamese speakers with LEP from Census Bureau summary statistics. Cantonese and Mandarin speakers with LEP were derived from 2011 to 2015 ACS PUMS 5-year estimates.^19^

We calculated PCP supply (the ratio of PCPs per 100,000 individuals with LEP), assuming that 100% of bilingual PCPs patients were language-concordant patients with LEP. Given that it is unlikely that language-concordant patients with LEP would comprise all patients in a self-reported bilingual PCP's practice, we performed two sets of sensitivity analyses. We could not use Medical Board survey data because the survey did not ask self-reported bilingual physicians to report the percentage of their patients who speak the same non-English languages.

For a sensitivity analysis, we assumed that a self-reported bilingual PCP's panel reflected the percentage of California's non-English-speaking population with LEP for each given language according to the 2011–2015 Census. We then conducted a second sensitivity analysis assuming that 22% of a self-reported bilingual physician's panel included language-concordant patients with LEP. This percentage was chosen based on a previously reported study, which found that 22% of visits for Spanish self-reported bilingual PCPs in a large group practice in California were language concordant in Spanish.^22^ Calculations were then repeated by region. All analyses were done using Stata Version 13.1 (College Station, TX). This study was reviewed and approved by the University of California Committee on Human Research (UCSF IRB).

## Results

Among the 139,222 physicians who renewed their licenses in California during the 2-year study period, 19,310 were active PCPs as per our inclusion criteria. Of these, 15,933 (83%) answered the language proficiency question and comprised the final sample for our analysis (Table 1).

**Table 1.** California Primary Care Physician Characteristics (*N*=15,933)

	California primary care physicians,^a^*N*=15,933	Spanish-speaking primary care physicians, *N*=5,203 (33%)		Cantonese-speaking primary care physicians, *N*=486 (3%)		Mandarin-speaking primary care physicians, *N*=986 (6%)		Tagalog-speaking		Vietnamese-speaking primary care physicians, *N*=671 (4%)	
								primary care physicians, *N*=956 (6%)			
	*N* (%)	*N* (%)	*p*	*N* (%)	*p*	*N* (%)	*p*	*N* (%)	*p*	*N* (%)	*p*
Age			0.011		<0.001		0.367		<0.001		0.088
<46	6,061 (38)	2,057 (40)		139 (29)		376 (38)		213 (22)		282 (42)	
46–60	6,258 (39)	1,965 (38)		209 (43)		403 (41)		424 (44)		243 (36)	
>60	3,614 (23)	1,181 (23)		138 (28)		207 (21)		319 (33)		146 (22)	
Male	8,583 (54)	2,769 (53)	0.252	288 (59)	0.015	558 (57)	0.077	436 (46)	<0.001	432 (64)	<0.001
Race/ethnicity^b^			<0.001		<0.001		<0.001		<0.001		<0.001
African American	429 (3)	159 (3)		0		— (<1)		— (<1)		0	
Asian Pacific Islander^c^	6,404 (43)	1,219 (25)		446 (94)		911 (95)		884 (94)		643 (97)	
Latino	1,419 (9)	1,371 (28)		— (<1)		— (<1)		— (<1)		0	
Other	536 (4)	200 (4)		— (1)		— (<1)		17 (2)		— (<1)	
White	4,184 (28)	1,376 (29)		0		0		— (<1)		0	
Two or more	— (<1)	— (<1)		0		0		— (<1)		0	
No response/decline to state	2,932 (18)	869 (17)		33 (7)		60 (6)		45 (5)		23 (3)	
U.S. medical graduate	9,109 (57)	3,502 (67)	<0.001	307 (63)	0.025	459 (47)	<0.001	73 (8)	<0.001	389 (58)	0.673
Specialty			<0.001		0.001		<0.001		<0.001		<0.001
Family Medicine	6,296 (40)	2,540 (49)		165 (34)		327 (33)		418 (44)		288 (43)	
Internal Medicine^d^	6,232 (39)	1,248 (24)		231 (48)		475 (48)		275 (29)		305 (45)	
Pediatrics	3,405 (21)	1,415 (27)		90 (19)		184 (19)		263 (28)		78 (12)	

^a^ Includes California Primary Care Physicians who have an active license, who work ≥20 h a week in direct patient care, who are no longer in training, and who responded to the question about language proficiency (15,933 out of 19,310 physicians answered the language question).

^b^ For categories with ≤1%, numbers are not presented to protect the confidentiality of providers.

^c^ Asian Pacific Islander: includes Chinese, Filipino, and Vietnamese.

^d^ Internal Medicine (includes General Practice and Geriatric Medicine).

Among PCPs who self-reported their language skills, 5,203 (33%) spoke Spanish, 486 (3%) spoke Cantonese, 986 (6%) spoke Mandarin, 956 (6%) spoke Tagalog, and 671 (4%) spoke Vietnamese (Table 1). While most Cantonese-, Mandarin-, Tagalog-, and Vietnamese-speaking PCPs self-identified as Asian Pacific Islander, PCPs of all racial/ethnic backgrounds reported Spanish proficiency. Similar numbers of Spanish-speaking PCPs self-identified as non-Hispanic white (29%), Latino (28%), and Asian Pacific Islander (25%). Eighteen percent of our sample declined to state ethnicity or left the measure blank. PCPs who reported speaking Tagalog and Mandarin were less likely to be U.S. medical graduates (8% and 47%, respectively) than PCPs who reported speaking Spanish, Cantonese, or Vietnamese. Overall, 57% of PCPs graduated from a U.S. medical school.

According to ACS PUMS 5-year estimates, in 2011–2015, there were 4.4 million Spanish-speaking, 152,779 Cantonese-speaking, 120,620 Mandarin-speaking, 266,692 Tagalog-speaking, and 319,841 Vietnamese-speaking individuals with LEP in California. During this time period, 12% of California's population consisted of Spanish speakers with LEP and 0.4%, 0.3%, 0.7%, and 0.9% of the population were composed of Cantonese, Mandarin, Tagalog, and Vietnamese speakers with LEP, respectively.

Table 2 describes the supply of PCPs for the entire Californian population and for the population with LEP by primary language spoken under the three assumptions regarding the percentage of patients with LEP on self-reported bilingual physicians' panels. When assuming that 100% of the patients of all self-reported bilingual physicians have LEP and that the physicians and patients speak the same non-English language, there is an adequate supply of PCPs who report speaking all 5 languages (117 PCPs per 100,000 LEP Spanish speakers, 318 per 100,000 for LEP Cantonese speakers, 817 per 100,000 for LEP Mandarin speakers, 358 per 100,000 for LEP Tagalog speakers, and 210 per 100,000 for LEP Vietnamese speakers) relative to the federal benchmark of 60–80 PCPs per 100,000 population.

**Table 2.** Supply of Primary Care Physicians for the Entire Californian Population and for Limited English Proficiency Speakers of the Five Target Languages

	Physicians per 100,000 individuals	Spanish-speaking physicians/100,000 LEP Spanish-speaking individuals			Cantonese-speaking physicians/100,000 LEP Cantonese-speaking individuals			Mandarin-speaking physicians/100,000 LEP Mandarin-speaking individuals			Tagalog-speaking physicians/100,000 LEP Tagalog-speaking individuals			Vietnamese-speaking physicians/100,000 LEP Vietnamese-speaking individuals		
		100%	22%	12%	100%	22%	0.4%	100%	22%	0.3%	100%	22%	0.7%	100%	22%	0.9%
Primary care	54^a^	117	26	14	318	70	1	817	180	2	358	79	3	210	46	2
Family Medicine	18	57	13	7	108	24	<1	271	60	<1	157	35	1	90	20	<1
Internal Medicine^b^	17	28	6	3	152	33	<1	394	87	<1	103	23	<1	95	21	<1
Pediatrics	9	32	7	4	59	13	<1	153	34	<1	99	22	<1	24	5	<1

Ratios presented for varying assumptions about the percentage of physicians' patients with LEP (assuming 100%, 22%, and the representative percentage of the California population with LEP in each language). Percentages of individuals in the California population with LEP: Spanish speaking=12%, Cantonese speaking=0.4%, Mandarin speaking=0.3%, Tagalog speaking=0.7%, and Vietnamese speaking=0.9%.

^a^ All primary care physicians included in this estimate, regardless of whether they answered the question about language proficiency or not. All physicians=19,310.

^b^ Internal Medicine (includes General Practice and Geriatric Medicine).

LEP, limited English proficiency.

However, if we assume that the percentage of each self-reported bilingual physician's patients with LEP and who speak the same non-English language is equal to the representation in the California population, the supply falls below the federal benchmark for all languages: Spanish (14 PCPs), Cantonese (1 PCP), Mandarin (2 PCPs), Tagalog (3 PCPs), and Vietnamese (2 PCPs) per 100,000 individuals with LEP. The supply is still below the federal benchmark for Spanish (26 PCPs) and Vietnamese (46 PCPs) if we assume, based on a previous study of a large group practice in California, that 22% of each self-reported bilingual physician's patients are with LEP and speak the same non-English language.

Table 3 demonstrates PCP supply by region for each of the study languages. While there is variation at the regional level, the same patterns emerge as seen with the statewide estimates. When one assumes that self-reported bilingual PCP panels include 100% concordant LEP patients, there is an adequate supply in all regions. However, the supply is increasingly inadequate under the various assumptions of the percentage of a PCP's patient panel with LEP.

**Table 3.** Supply of Primary Care Physicians for the Entire Californian Population and for Limited English Proficiency Speakers of the Five Target Languages by Region

	Physicians per 100,000 individuals	Spanish-speaking physicians/100,000 LEP Spanish-speaking Individuals			Cantonese-speaking physicians/100,000 LEP Cantonese-speaking individuals			Mandarin-speaking physicians/100,000 LEP Mandarin-speaking individuals			Tagalog-speaking physicians/100,000 LEP Tagalog-speaking individuals			Vietnamese-speaking physicians/100,000 LEP Vietnamese-speaking individuals		
		100%	22%	12%	100%	22%	0.4%	100%	22%	0.3%	100%	22%	0.7%	100%	22%	0.9%
Bay Area	56	207	46	25	275	61	1	898	198	3	185	41	1	155	34	2
Central Coast	41	109	24	13	827	182	3	992	218	3	110	24	<1	448	99	7
Central Valley/Sierra	42	89	20	11	922	203	4	4,101	902	12	910	200	6	447	98	7
Inland Empire	32	66	15	8	262	58	1	1,042	229	3	783	172	5	458	101	7
Los Angeles	45	103	23	12	307	68	1	566	125	2	381	84	3	282	62	4
North	35	233	51	28	1,460	321	6	2,970	653	9	1,404	309	10	239	53	4
North Valley/Sierra	44	156	34	19	325	72	1	1,655	364	5	438	96	3	204	45	3
Orange	46	130	29	16	1,319	290	5	1,051	231	3	389	86	3	177	39	3
San Diego	43	154	34	18	245	54	1	713	157	2	147	32	1	152	33	2
South Valley/Sierra	34	69	15	8	1,190	262	5	51,995	11,439	156	1,290	284	9	578	127	9

Ratios presented for varying assumptions about the percentage of physicians' patients with LEP (assuming 100%, 22%, and the representative percentage of the California population with LEP in each language). Percentages of individuals in the California population with LEP: Spanish speaking=12%, Cantonese speaking=0.4%, Mandarin speaking=0.3%, Tagalog speaking=0.7%, and Vietnamese speaking=0.9%.

## Discussion

Estimates of access to language-concordant PCPs for individuals with LEP are sensitive to assumptions about the proportion of patients with LEP in a PCP's panel. Supplies of self-reported bilingual PCPs are adequate if we assume 100% of patients treated by each provider are patients with LEP, but significant gaps emerge if we assume lower percentages of PCP panels comprised patients with LEP. If PCPs treat a proportion of patients with LEP relative to the population with LEP in California, the supply is inadequate for all groups studied. Although the exact percentages of bilingual physicians' patients who are language concordant in a language other than English are unknown, the percentage is probably <100% for most PCPs, given the shortage of PCPs in California.^20,23^ The percentage is also probably greater than the proportion of California's population with LEP, given that previous research suggests that physicians who practice in areas with high concentrations of individuals with LEP are more likely to have self-reported non-English language proficiency.^14,15^

Our study may overestimate primary care access to language-concordant providers for patients with LEP because we do not have any information about whether patients with LEP have health insurance or the type of health insurance they have. Patients with LEP are more likely to be uninsured and are overrepresented among Medicaid beneficiaries.^24^ Prior studies have shown that access may be even more limited for Spanish speakers when type of insurance is considered.^13^

We further found noteworthy racial and ethnic differences among physicians who self-reported proficiency in the five non-English languages. The greater racial and ethnic diversity of Spanish-speaking physicians, compared to physicians who speak the four Asian languages, may reflect increasing number of Spanish immersion curriculums in medical schools, prioritization of Spanish language skills in medical school and residency admission, hiring preferences that value additional language abilities, or responses of individual PCPs to the growing Latino population in California.^9,14,25^ Similar to prior studies, we also found that PCPs reporting non-English language skills were more likely to be international medical graduates, with the exception of physicians who speak Spanish. In our study, while there was some regional variation in PCP supply, numbers remained low when accounting for the population with LEP in each region. While physicians may self-select to certain areas to improve language concordance, other structural forces likely play a larger role in choosing a region for a PCP's practice (cost of living, housing, educational system, etc).

Our study has a few limitations. We rely on self-reported provider proficiency, which may not accurately reflect provider fluency and may overestimate the number of providers proficient in the five target languages. Furthermore, the survey question on self-reported language proficiency is binary, thus assuming that a provider either does or does not speak a language. In practice, there are gradations of proficiency, as well as context-dependent factors, which affect whether providers rely on personal proficiency or not in a given situation (such as the availability of interpreters and sensitivity or complexity of a given clinical conversation). A binary system of defining non-English proficiency may significantly overestimate the number of providers who are proficient and thus the supply of language-concordant PCPs. This may be particularly true for self-reported Spanish-speaking providers, who include providers from a greater mix of racial/ethnic backgrounds and fewer IMGs. Self-report may overestimate the linguistic skills of providers who are not native speakers of a non-English language. While there have been recent efforts to certify physicians for use of a non-English language in routine clinical practice, certification is still not widespread.^26^ In addition, cultural sensitivity, separately from language concordance, may affect the patient-provider relationship.^27^ For example, a provider may be proficient in a patient's language, but may not understand cultural factors that affect how a patient describes his or her condition or receptivity to treatment options. However, providers' cultural background or their cultural understanding of their patients is beyond the scope of this study.

Other limitations include the number of nonresponders to the question on self-reported language proficiency (17% of eligible PCPs). These individuals were excluded from the overall denominator for our analyses, which could lead to an inaccurate assessment of the percentages of self-reported bilingual PCPs. In addition, we did not have data on the percentages of self-reported bilingual physicians' patients with LEP. Our sensitivity analyses may not reflect the actual percentages of patients with LEP in their practices. In particular, our estimates of supply if 100% of patients with LEP in a PCP's panel are language concordant are most certainly an overestimate.

## Conclusion

Our study of California, one of the most populous states in the United States, provides a foundation for further analyses of PCP language capacity. In particular, it is the first study to examine proficiency in individual Asian languages (as opposed to lumping them together) and provides an updated estimate for PCP supply for Spanish-speaking patients with LEP.^13,16,17^ We believe that these results may reflect national trends with important implications for health workforce projections and planning beyond California. Our study also highlights important gaps in the literature and in current data collection methods. While many factors influence access to PCPs (e.g., geographical distance, insurance, and panel availability), future studies are also needed to determine the degree of language matching that currently occurs in primary care practice between bilingual providers and non-English-speaking patients. This information could be collected among PCPs who report non-English language proficiency, such as with the addition of this question in the Medical Board of California survey or through a national physician survey, such as the National Ambulatory Medical Care Survey. In addition, since there are gradations in language proficiency, efforts should be made to improve the precision of data collection on physician non-English language skills, which would allow for more accurate measurement and more robust future studies.

As the diversity of languages represented increases, there will be a continuing need to ensure access and adequate training of interpreters in different languages (beyond the five target languages included in this study). However, prior literature has demonstrated that, while interpreters often helped in the provision of health education, patients who used interpreters reported lower interpersonal care and patient satisfaction than patients who had language-concordant providers.^10^ Furthermore, another study found that patients who used interpreters were more likely to have remaining questions about their care or mental health after a clinic visit than patients who had language-concordant providers.^28^ Team-based care, including trained interpreters, nurses, medical assistants, and other members of the clinical care team, may further help close the linguistic gap between providers and their patients with LEP.

Other ways that California and other states can meet the needs of patients with LEP include ensuring that physicians who use non-English languages in clinical encounters have sufficient proficiency so that they do not introduce safety risks. Medical schools might also consider non-English language skills as another factor in the decision for admission to their schools. Some have called for programs to reward physicians who achieve medical competency in another language and for health systems to routinely collect provider non-English language proficiency data, to facilitate patient-provider language concordance.^9^ These approaches merit consideration, but, because they are likely to have significant implementation costs, further studies should assess whether they improve outcomes for patients with LEP and prove cost-effective.

## Abbreviations Used

ACS: American Community Survey
BUILD: Building Infrastructure Leading to Diversity
LEP: limited English proficiency
PCP: primary care physician
PUMS: Public Use Microdata Sample
